# Hyperspectral Shortwave Infrared Image Analysis for Detection of Adulterants in Almond Powder with One-Class Classification Method

**DOI:** 10.3390/s20205855

**Published:** 2020-10-16

**Authors:** Mohammad Akbar Faqeerzada, Santosh Lohumi, Geonwoo Kim, Rahul Joshi, Hoonsoo Lee, Moon Sung Kim, Byoung-Kwan Cho

**Affiliations:** 1 Department of Biosystems Machinery Engineering, College of Agriculture and Life Science, Chungnam National University, 99 Daehak-ro, Yuseong-gu, Daejeon 34134, Korea; akbar.faqeerzada@gmail.com (M.A.F.); santosh.sanny123@gmail.com (S.L.); rahul.joshi98@yahoo.com (R.J.); 2 Environmental Microbial and Food Safety Laboratory, Agricultural Research Service, United States Department of Agriculture, Powder Mill Road, BARC-East, Bldg 303, BARC-East, Beltsville, MD 20705, USA; moon.kim@ars.usda.gov; 3 Department of Biosystems Engineering, College of Agriculture, Life & Environment Science, Chungbuk National University, 1 Chungdae-ro, Seowon-gu, Cheongju, Chungbuk 28644, Korea; hslee202@chungbuk.ac.kr; 4 Department of Smart Agriculture System, College of Agricultural and Life Science, Chungnam National University, 99 Daehak-ro, Yuseong-gu, Daejeon 34134, Korea

**Keywords:** food adulteration, almond powder, hyperspectral imaging, shortwave infrared, spectral analysis, one-class classification

## Abstract

The widely used techniques for analyzing the quality of powdered food products focus on targeted detection with a low-throughput screening of samples. Owing to potentially significant health threats and large-scale adulterations, food regulatory agencies and industries require rapid and non-destructive analytical techniques for the detection of unexpected compounds present in products. Accordingly, shortwave-infrared hyperspectral imaging (SWIR-HSI) for high throughput authenticity analysis of almond powder was investigated in this study. Two different varieties of almond powder, adulterated with apricot and peanut powder at different concentrations, were imaged using the SWIR-HSI system. A one-class classifier technique, known as data-driven soft independent modeling of class analogy (DD-SIMCA), was used on collected data sets of pure and adulterated samples. A partial least square regression (PLSR) model was further developed to predict adulterant concentrations in almond powder. Classification results from DD-SIMCA yielded 100% sensitivity and 89–100% specificity for different validation sets of adulterated samples. The results obtained from the PLSR analysis yielded a high determination coefficient (R^2^) and low error values (<1%) for each variety of almond powder adulterated with apricot; however, a relatively higher error rates of 2.5% and 4.4% for the two varieties of almond powder adulterated with peanut powder, which indicates the performance of quantitative analysis model could vary with sample condition, such as variety, originality, etc. PLSR-based concentration mapped images visually characterized the adulterant (apricot) concentration in the almond powder. These results demonstrate that the SWIR-HSI technique combined with the one-class classifier DD-SIMCA can be used effectively for a high-throughput quality screening of almond powder regarding potential adulteration.

## 1. Introduction

Authentication of powdered food products is becoming increasingly important owing to growing numbers of food fraud incidents, particularly in high value-added products. Owing to their multiple unique features and high nutritional value, almonds are one of the most popular nuts consumed worldwide. They are consumed either as a whole nut or in powdered form in a variety of processed foods, particularly in baking and confectionery products [1]. Although all food products can potentially tempt to adulteration, it estimated that around 22% of foods are adulterated every year [2]. The powder foods are more vulnerable for being adulterated by adding the cheap materials of similar texture and color to the original food product to increase the quantity [3]. To highlight, one of the most notorious food adulteration events was the adulteration of melamine in baby milk, which caused six children to die and several thousand to be hospitalized [4]. Another incident by the adulteration of paprika with colored agents caused 60 people to be hospitalized [5].

Because of its high price and worldwide consumption, almond powder is susceptible to economic adulteration through the addition of less-expensive nut powders. The most common adulterant of almond powder is an apricot powder, owing to its lower cost, its close similarity in color, texture, and odor to almond powder, and other physiochemical characteristics [6]. Secondly, the peanut powder, as an adulterant, has a chemical composition similar to that of almond powder and is less expensive [7]. Although almond powder adulteration with apricot powder does not have detrimental effects on human health, it reduces the nutritional value of the product. However, peanut as an adulterant can pose a threat to people allergic to peanuts. Among the nuts allergy to peanuts is a potentially life-threatening condition due to the induction of anaphylaxis, which is a severe, potentially life-threatening reaction [8,9]. This serious allergic reaction disease affects 150,000 Americans a year [10]; approximately, it causes 100 deaths in the U.S. due to peanuts allergy [11].

Recently, dilution of curry sauces with peanuts caused three deaths in the UK. Despite the sauce purporting to be peanut-free, investigations linked the cause to peanut allergies [12]. As a result, the food manufacturer and wholesaler was ordered to pay 18,000 Euros after it was found guilty of potentially fatal food adulteration, supplying it to another wholesaler company. The investigation found the food manufacturer was supplied ground peanuts, even though pure almond powder was requested by its clients running a restaurant in the UK. Peanut allergy is becoming increasingly commonplace, with recent studies indicating that the rate has doubled over the past five years in Europe and the USA [13]. Therefore, it is important to develop effective analytical techniques for quality and authenticity screening of almond powder to detect potential adulterants (apricot and peanut).

Various studies have used conventional techniques combined with chemometric analysis for the detection of known adulterants in powdered food. Adulteration of almond with apricot was detected using chromatographic fingerprinting with multivariate data analysis [6]; this was also the case in other studies conducted for the qualitative and quantitative analysis of almond powder adulterated with peanuts using multi-elemental fingerprinting combined with chemometrics [7]. The chromatographic techniques are usually accurate, sensitive, and reliable, but are generally time-consuming, requiring sample preparation and the use of chemicals for extracting the required information.

Several spectroscopic techniques have recently been used, such as near-infrared, infrared, and Raman spectroscopy, to detect a variety of adulterants in various powdered food products. Fourier transform near-infrared (FT-NIR) and Fourier transform infrared (FT-IR) spectroscopy have been used for the adulteration of onion powder with cornstarch [14]. Other examples include the identification of paprika powder adulteration with Sudan dye, which showed high accurate detection performance [15], and the application FT-Raman and FT-IR spectroscopy for detecting Metanil Yellow adulterant in turmeric, in which FT-Raman, detecting a low percent of concentration (1%), gave better results than FT-IR [16]. These spectroscopic methods provide high sensitivity and specificity, are nondestructive and less time-consuming (sample preparation and measurement being single sample-based). As reported by the aforementioned studies, they achieve a high accuracy, however, the main drawback is that a large number of specimens or bulk amounts of samples cannot be analyzed rapidly, which is typically the case in food authenticity analyses. Thus, a rapid technique with the ability to inspect food material in a large quantity is in high demand.

The main advantage of hyperspectral imaging (HSI) technology is that it can obtain spatial and spectral information from objects simultaneously, which provides chemical images rapidly with the application of spectral analysis. The HSI technique make sample analysis more convenient and faster since a large number of samples can be analyzed at the same time, as compared to the single sample technique used for the spectroscopic techniques described above [17]. Significant focus has been directed toward the utilization of the HSI technique as an effective tool for powdered food authenticity analysis. Some of the studies showed good performance for the detection of food adulteration, such as peanut and walnut adulteration in wheat flour [18], melamine adulterant in milk powder [19], wheat, cassava, and corn flour in organic Avatar wheat flour [20], and peanut traces in wheat flour [21]. However, the majority of these studies have focused on target-based methods, such as partial least square regression (PLSR) and spectral similarity measure methods [22], applying an independent component analysis (ICA) method for processing NIR hyperspectral images to detect trace amounts of peanut in wheat flour. ICA is a powerful signal processing technique applicable to the blind source separation problem. This technique is particularly effective when there are pure pixels of adulterant material in the hyperspectral image; however, it is less sensitive when the pixels in the hyperspectral image attain mixed features involving both the food and adulterant powders, which is the case for almond powder adulteration with apricot and peanut powder.

According to the abovementioned reports, the analysis was based on target detection that can predict the contaminant with a high accuracy using hyperspectral imaging systems. Targeted models can be developed for a specific compound that is present in specific adulterants; however, for samples mixed with adulterants that are unknown to the model, the adulterants will evade detection. Thus, the development of rapid, real-time, and accurate models based on non-targeted tools are widely desired for the validation of food materials to ensure public health and increase food reliability.

We investigate the feasibility of non-targeted detection of almond powder adulteration using a one-class classifier analysis method in this study. Data-driven soft independent of class analogy (DD-SIMCA) is a one-class classification technique that only models the target class. In the classification step, a new sample may or may not belong to the target class. Any sample out of the threshold boundary decided during model development is considered an external sample (adulterated in our case). This type of one-class classification method appears to be more effective than the previously mentioned conventional classification techniques, particularly for adulteration analyses. However, the accepted boundaries for pure samples can be defined by the features of the pre-selected training dataset. A recent study compared the performance of the PLS discriminant analysis (PLS-DA) method and DD-SIMCA for an authentication problem, and DD-SIMCA achieved more reliable results [23,24,25]. Regarding non-targeted authentication of food materials, various studies have proposed the qualitative analysis of powdered-form products using a one-class classification technique to ensure food safety, such as the detection of quinoa flour adulteration using FT-MIR spectroscopy combined with chemometrics based on targeted and non-targeted detection. Examples of this include SIMCA with an accuracy of 100% for sensitivity and specificity [26], and NIR spectroscopy and SIMCA used for the classification of commercial milk powder authentication using 11 potential adulterants, which presented 100% sensitivity based on the specificity according to the adulterant [27]. In another study for the nondestructive identification of native egg, near-infrared spectroscopy and data driven-based class modeling were used, demonstrating a sensitivity of 100% and specificity of 93–100% [28]. The authors used attenuated total reflectance Fourier-transform mid-infrared spectroscopy [29] to analyze and detect adulterants in grape nectars, resulting in a sensitivity and specificity close to 100% overall; in addition, an improvement in the health of tomato seeds was achieved using an image-based classification method that presented over 97% accuracy [30].

The aforementioned studies have widely contributed to the detection of adulterated food materials, and potentially promoted nondestructive applications for the authentication of food products combined with non-targeted detections. Furthermore, we reported the feasible detection of adulterated almond powder with apricot and peanuts based on the using FT-NIR and FT-IR spectroscopy for nondestructive authentication of food materials. Both instruments demonstrated a good potential for the detection of adulterated almond powder with a high sensitivity (97–100%), high specificity (93–100%), and a 90–100% total accuracy [31]. Regardless of this, non-targeted detection classifiers are typically applied to spectroscopic data analysis, however they have been used mostly in the case of the point-based measurements for small sample amounts. Even the collected spectral data cannot present a similar pattern for all samples, particularly if the samples are chemically heterogeneous, as in the case of adulterated powdered food samples. Thus, in this study, SWIR hyperspectral imaging technique combined with DD-SIMCA data analysis was explored to detect unknown food adulterants in different varieties of almond powder. Quantitative analysis of food adulteration has a crucial role in providing information related to adulterant quantity for international trade and human health. Therefore, subsequently, a partial least square regression (PLSR) technique was used for the quantitative analysis of almond powder adulterated with apricot and peanut powder.

The main purpose of this study was to develop a high-throughput non-targeted detection analysis model for the quality and authenticity analysis of almond powder. The second objective was to build an effective and reliable model for the quantitative analysis of adulterant concentrations in almond powder based on hyperspectral imaging data, which can further be applied to the quantitative authenticity analysis of different varieties of almond powder. Within these contexts, we focused on the development and validation of a multivariate analysis model for both qualitative and quantitative determination of adulteration in mixtures of almond/apricot and almond/peanut powder.

## 2. Materials and Methods

### 2.1. Sample Preparation

Two varieties of almond powder and apricot powder (Ograe Granola) were paid commercially from a reputable food manufacturing plant (Agriculture Company Neulgreen) in Daejeon, South Korea, and peanut samples were obtained from local grocery stores. The samples were ground and stored at room temperature (20–22 °C) for a day before the experiments were conducted. Since differences in sample particle size can add artifacts in the spectra [32], all samples were first sewn using a 250 μm mesh screen to achieve same particle sizes of almond powder and adulterant materials. The almond powder was mixed with the different concentration of apricot adulterants from 0% to 50% with the increment of 5%. The second variety of almonds was processed in the same way as the first variety, but the different concentrations were used (0%, 7%, 15%, 22%, and 30%). This second variety was of different geographical origin than the first, possibly having a slightly different chemical composition. All samples weighed 7 g each. The sample mixture for almond/peanut powder was set in the same way with the almond/apricot powder mixture. Each mixture was blended manually and then transferred to a vial with a snap-cap. Additional mixing was achieved by putting the filled vials in a high-speed shaker (Vortex-Genie 2, Scientific Industries, Inc., model G560, Bohemia, NY, USA). 

Each mixed sample was divided into five sub-samples, packed into a custom-built, black- colored plate (25 holes: Each hole measuring 25 mm in diameter and 5 mm deep). Besides, two plates (50 sub-samples) of pure almond of each variety were also scanned to develop a calibration model for DD-SIMCA. Arranged sample holders were filled without compressing the powder samples and leveled across the top using a rod to smooth the surface of the sample and remove any excess powder.

### 2.2. Hyperspectral Imaging System (HIS)

**Figure 1:**
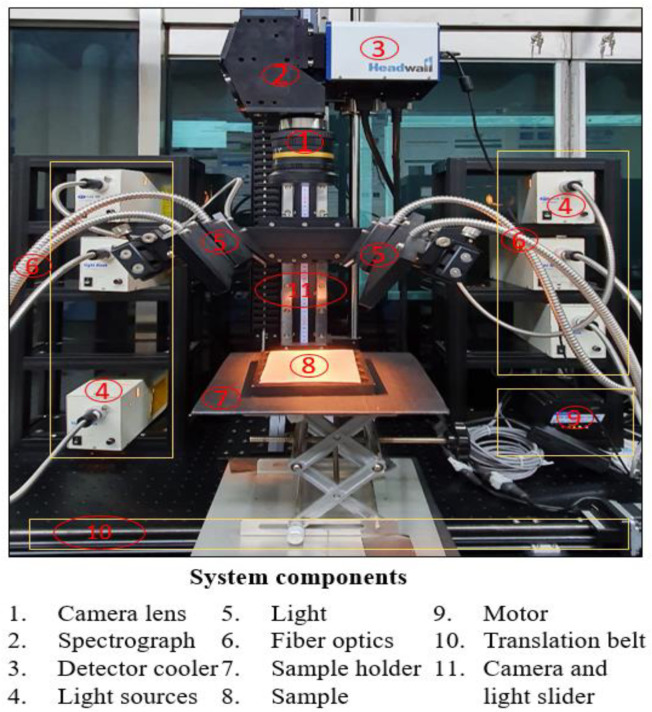
Hyperspectral imaging system and major components.

A line-scan hyperspectral imaging system in a short-wave infrared spectral range was used to perform fast and non-destructive screening of almond powdered samples. The system consisted of a line scan image spectrograph (Headwall Photonics, Fitchburg, MA, USA) with a spectral range of 900–2494 nm and 5.8 nm spectral resolution mounted onto a mercury cadmium telluride (MCT) detectors for detecting the back-reflected radiation from the sample. The illumination sources consisted of six 12 V, 100 W diffused tungsten-halogen lamps (Light Bank, Ushio INC., Tokyo. Japan) placed at equal distances from each other. A sample holder, a stepper motor connected to a personal computer, and a linear stage were combined to move samples under the line scan imaging system. In order to cover the complete spatial range of the sample, the distance between camera and lens was adjusted to 20 cm. The hypercube dimensions consist of 324 pixels of a fixed spatial direction, n pixels of unfixed spatial direction depending on the length of the sample, and 275 pixels (wavebands) in the spectral direction. Figure 1 shows the schematic of the hyperspectral imaging system used for the experiments. 

### 2.3. Image Calibration

The spectral intensity of the measured raw hyperspectral images was converted to relative reflectance intensity through Equation (1). Due to the quantum effect of the utilized camera, the uncorrected radiance for the different systems, even for the same system used at different times, can be varied with the same sample taken under the same conditions [33]. Hence, reflectance calibration was critical to ensure the reliability and acceptability of the spectral information of the hyperspectral image data and the system. The raw hypercube can be calibrated by the following reflectance model based on the reference images of dark current and white plate of Teflon.

(1) $$X_{\mathrm{cal}}=\frac{X_{\mathrm{raw}}-X_{\mathrm{dark}}}{X_{\mathrm{ref}}-X_{\mathrm{dark}}}$$

The calibration image X_cal_ was derived by the raw hyperspectral image X_raw_, dark current image X_dark_, and white reference image X_ref_.

### 2.4. Spectral Extraction

**Figure 2:**
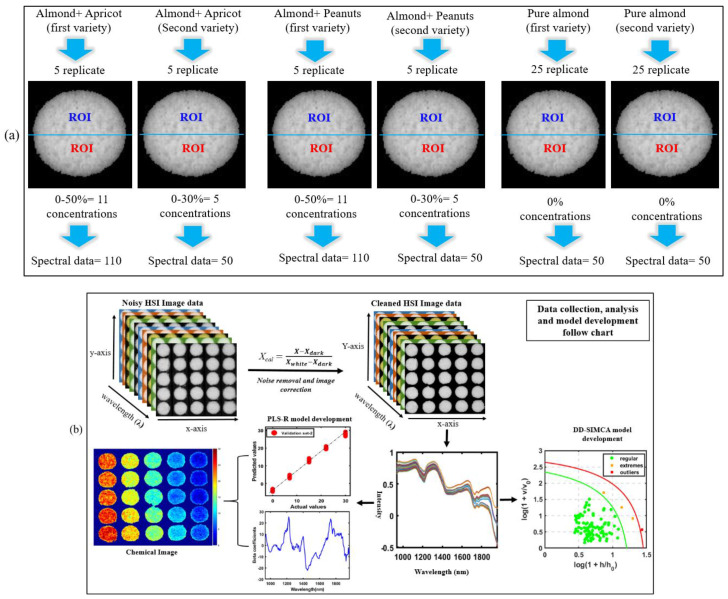
(**a**) Spectral data division and extraction process from the region of interest (ROI) of the samples and (**b**) flow chart of hyperspectral image correction and data analysis.

The background from the calibrated hyperspectral image was removed by use of a threshold value calculated by the average value of the background and powder sample pixels. The spectral data in between 935 nm and 1965 nm were extracted from the region of interest (ROI) in the processed hyperspectral images. Each concentration subset (0–50% each with two replications of five samples) of imaged samples was divided into two equal parts for extraction of information, from which 110 spectral data (10 for each concentration) for the first variety of almond were extracted. The same method was used for obtaining the spectral data from the second group of samples (second variety of almond powder) with adulterant concentration of 0%, 7%, 15%, 22%, and 30%. For the remaining two plates of measured pure samples of both varieties (each with 25 subsets), each was divided into two halves and each half was averaged, producing a total of 100 spectral data (2 varieties × 25 subsets × 2 halves) that were extracted using pure almond powder to develop the DD-SIMCA calibration model. For more simplicity of the data interpretation, Figure 2a indicates the spectral data division and extraction process from the ROI of the samples; and Figure 2b indicates the image correction and further shows the data analysis strategy.

### 2.5. Data Analysis

#### 2.5.1. Spectral Pre-Processing

**Figure 3:**
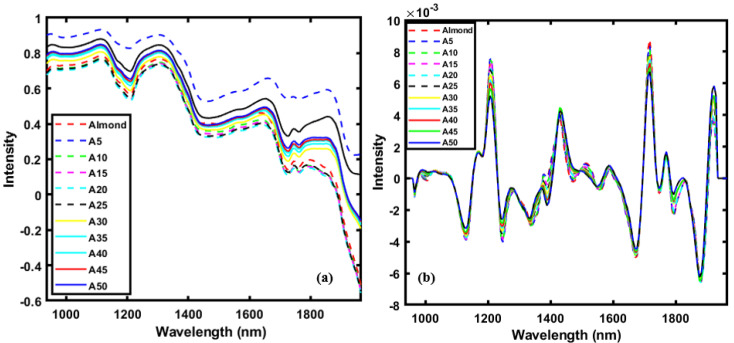
(**a**) Raw and (**b**) SG-2nd derivative pre-processed mean spectra of almond powder samples with different adulterant (apricot powder) concentrations.

Spectral pre-processing is applied to correct random noise in spectra, length variation of the light direction, and instrument-generated light scattering. Hence, it is necessary to pretreat the obtained spectral data using appropriate mathematical analysis to emphasize the valuable information embedded in the sample while eliminating undesired variations from the data [34]. In this study, several spectral pre-processing methods, such as the normalization (minimum, maximum, and range normalization), smoothing, regular normal variate (SNV), multiplicative scatter correction (MSC), and Savitzky-Golay (SG) first and second derivative methods of were used. Figure 3a,b show the raw spectral data, and the SG-2nd derivative plots demonstrate the differences after pre-processing almond/apricot powder sample data. From Figure 3a, it can be seen that the raw spectra are highly affected by the baseline effect, and thus showing no correlation between the adulterant concentration and spectral peak intensities. However, the application of the SG-2nd derivative preprocessing method corrected the baseline effect and the adulterant concentration-related variation can be seen throughout the whole spectral range in Figure 3b.

#### 2.5.2. DD-SIMCA

Spectroscopic data consists of a large number of variables that can be difficult to be interpreted without multivariate analytical methods and tools. DD-SIMCA, which is a single-class or targeted-class classification technique was used in this study. As aforementioned, the DD-SIMCA model was developed initially with a calibration set consisting of 100 spectral data of two varieties of pure almond powder. The first process was to decide the number of factors for each model. By evaluating the sensitivity (SEN) of the developed model, the required number of factors were determined. The DD-SIMCA projection is based on the calculating of score distance (SD, leverage) and the orthogonal distance (OD, residual), which defines two boundaries of decision. Principal component analysis (PCA) method was used to determine two levels or borders of decision boundaries which evaluate the performance of the sample classification. The outlier was identified with the significance level of 0.01, and the acceptance boundary was decided by the Chi-square distribution. The validation of the developed model was performed using two sets of almond/apricot and two sets of almond/peanut powder mixtures. Table 1 includes a description of the statistics for the calibration and validation data sets.

The entire process of the DD-SIMCA approach was carried out using MATLAB software (MathWorks, Natick, MA, USA) [35].

The performance of the developed DD-SIMCA model was evaluated with sensitivity, specificity, and total classification accuracy. Sensitivity and specificity were defined by the following Equations (2) and (3) respectively, and the total classification accuracy was the rate of correctly detected sample number among the entire number of samples as indicated in the Equation (4).

(2) $$Sensitivity=\left(\frac{TP}{TP+FN}\right)*100=\left(\frac{Number\;of\;detected\;pure\;samples}{Number\;of\;total\;pure\;samples}\right)*100$$

(3) $$Specificity=\left(\frac{TN}{FP+TN}\right)*100=\left(\frac{Number\;of\;detected\;adulterated\;samples}{Number\;of\;total\;adulterated\;samples\;}\right)*100$$

(4) $$Accuracy=\left(\frac{TP+TN}{TP+FP+FN+TN}\right)*100=\left(\frac{Number\;of\;correctly\;detected\;samples}{Number\;of\;total\;samples\;}\right)*100$$

where, in this work, True Positive (TP), a positive response for a positive one indicates the number of correctly detected pure samples; False Positive (FP), a positive response for a negative one means the number of pure samples predicted as adulterated ones; False Negative (FN), a negative response for a positive one indicates the number of adulterated samples predicted as pure ones; True Negative (TN), a negative response for a negative one means the number of correctly detected adulterated samples.

In addition, the calibration model created a plot displaying the position of samples that were identified as regular, extreme, and outlier. An optimal number of factors could be determined when the number of extreme samples was minimal in the model. To build a focused model, outliers were completely eliminated. 

#### 2.5.3. Partial Least Square Regression (PLSR)

PLS regression is a multivariate analysis technique that generalizes and combines features based on principal component analysis and multiple regression [36]. It is widely used as a multivariate calibration method for processing large amounts of data to predict the behavior of dependent variables based on large datasets of independent variables [37,38]. The PLSR model depends on consideration of the *X* and *Y* variables in a designed matrix, in which the linear relationship between the *X* and *Y* variables enables the model to predict the components in the *X* variables [39]. The model is defined as follows:

(5) $$X=TP^{T}+E$$

(6) $$Y=UQ^{T}+E$$

According to the model, *X* and *Y* are the independent and dependent variables, respectively, *T* and *U* denote score matrices, $P^{T}$ and $Q^{T}$ represent *X* and *Y* variable loading in the matrices, and *E* is the error in the matrix. The *X*-axis indicates the extracted spectral data from powder samples and the *Y*-axis represents the respective percentage values of adulteration.

For the prediction of adulterant concentration in an almond powder sample, the data matrix of the PLS model consisted of *X* matrix spectral data of the powder samples with different adulterant concentrations, and the *Y* vector represented the adulterant concentrations. The calibration model was first developed with one variety of almonds adulterated with different concentrations of apricot or peanut powder. The performance of the PLS model was then validated with two external datasets consisting of different concentrations of adulterant mixed with either the same or the second (different) variety of almond powder.

Selecting the number of latent variables (factors) is critical to avoid over- or under-fitting to develop a robust model. In this study, the optimal number of factors was selected based on the minimum value of the root-mean-square (*RMS*) method during the cross-validation (leave-one-out) process by applying Equation (7). The prediction efficiency of the PLS model was assessed using coefficients of determination for calibration and prediction ($R_{c}^{2}$ and $R_{v}^{2}$), respectively, and standard errors of calibration and prediction (SEC and SEV), respectively:

(7) $$RMSE=\frac{1}{Z}\sqrt{\overset{2}{\underset{i=1}{\sum }}{\left(y_{i-}{\hat{y}}_{i}\right)}^{2}}$$

where $y_{i}$ is the actual reference value of samples, ${\hat{y}}_{i}$ is the predicted value from the PLSR, and *Z* is the number of predictions.

#### 2.5.4. Chemical Mapping Based on the Image

One major advantage of hyperspectral imaging is its capability to create a chemical image of component distributions from simultaneous measurement of spectral and spatial data. The novel benefit of the chemical image is that it provides a measurement of different parameters of a chemical component, sample to sample, or even with the same sample, at each pixel location [40,41,42,43]. The PLSR beta coefficient was used to develop a chemical image of adulterated almond powder with mixed substances. In this process, the hyperspectral image was transformed into a 2D matrix and multiplied by the PLS regression coefficient. The obtained 2D matrix was folded back into the 3D image, and by summing the corresponding pixels of all band images, the generated PLS (chemical) image was used to facilitate the visualization of different adulteration concentrations in the samples. The final chemical images were obtained by applying the following equation:

(8) $$Chemical\;Image=\overset{n}{\underset{i=1}{\sum }}I_{i}R_{i}+C$$

where *I_i_* represents the measured hypercube image at the *i*th band, *R_i_* is the value of beta coefficient derived from the developed model, and *C* is a constant value. MATLAB software (MathWorks, Natick, MA, USA) was used to conduct all image processing and statistical analyses. 

## 3. Results and Discussion

### 3.1. Spectral Profile of Almond and Adulterants

Almond, apricot, and peanuts are a rich source of lipids and protein with different amounts of composition. Among these nuts, the peanuts with having 66% lipids and 21% protein composition, followed by the almonds with 53–61% lipids and 19% protein [44], while the apricot is consisting of 49–56% of lipids and 22–29% of dietary protein [45]. Then, spectral profiles of the lipid and protein were obtained to analyze the spectrum characteristics of their major chemical component.

**Figure 4:**
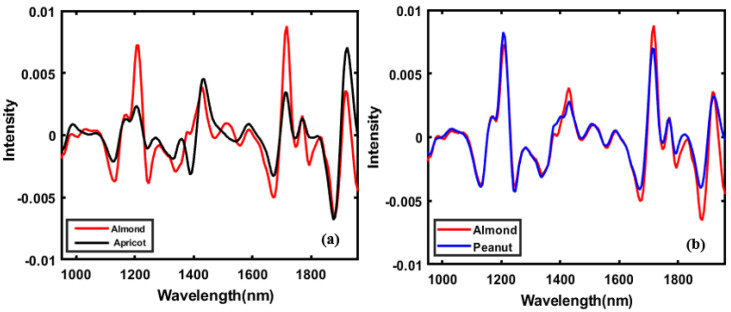
Mean spectra of almond powder and adulterants pre-processed using SG-second derivatives: (**a**) Almond and apricot, and (**b**) almond and peanut.

Figure 4a,b shows the mean spectra of almond and adulterants pre-processed using SG-2nd derivatives in the range of 935 to 1965 nm. From a visual comparison of the spectral data plotted in Figure 4a, apricot and almond spectra show greater dissimilarity than almond and peanut spectra. Thus, this dissimilarity in spectral data may lead to a better classification result for apricot-adulterated almond powder. Figure 4b presents the mean spectra of almond and peanut powders, which had many similar peaks, reflecting the similarity in chemical compositions over different ranges.

Based on previous studies, the peaks in the ranges of 1165, 1395, 1692, and 1734 nm are related to the lipid bands, attributable to the C−H (−CH) second overtone stretching band [21], and the lipids spectral band shows the higher concentrations in the almonds as shown the higher peaks in Figure 4a compared to the apricot, due to having a higher amount of oil than apricots. Meanwhile, in Figure 4b, in the same peaks at 1165 and 1395, peanuts shows higher peaks than almond, due to the representation of more amount of peroxide value (meq O_2_/kg oil) in peanuts [46]. Additionally, the peaks in 1200 nm corresponding to the protein displayed higher peaks in peanuts spectral signature compared to the almond and apricots, due to consisting of more protein in the peanuts [22].

In the same pattern, the 1690 nm peaks [47] and 1800 nm represented the existence of protein in the nuts [48], that peanuts spectral shows slightly higher peaks compared to other nuts. The peaks at 995 nm were thought to be caused by the N-H second overtone associated with peptides and proteins [49]. In the peanuts, the peaks at 1395 nm and 1734 nm belonged to CH2 bonds. They caused, respectively, the C–H stretch second overtone, the combination of 2C–H and C–H deformation, and the C–H first overtone. These peaks reflected the absorbance of long-chain fatty acids in the chemical composition of the nuts. That shows slightly higher peaks in the almond compared to other nuts, which might be due to a higher amount of long-chain fatty acids in almond seeds. The peaks at 1450 and 1940 nm were caused by the O–H stretch bond first overtone and the combination of an O–H bond stretch and deformation, respectively [21].

Thus, by interpreting the spectral features of almond, peanuts, and apricots powder, it can be concluded that the main differences lie in the spectral regions representing the lipid, protein, and carbohydrates. It should be noted that the main chemical or nutrient compositions of almonds, peanuts, and apricots are total lipid (fat), protein, carbohydrate, total dietary fiber, and sugar [50,51].

### 3.2. Class Modeling Construction Based on DD-SIMCA and Validation Performance

**Figure 5:**
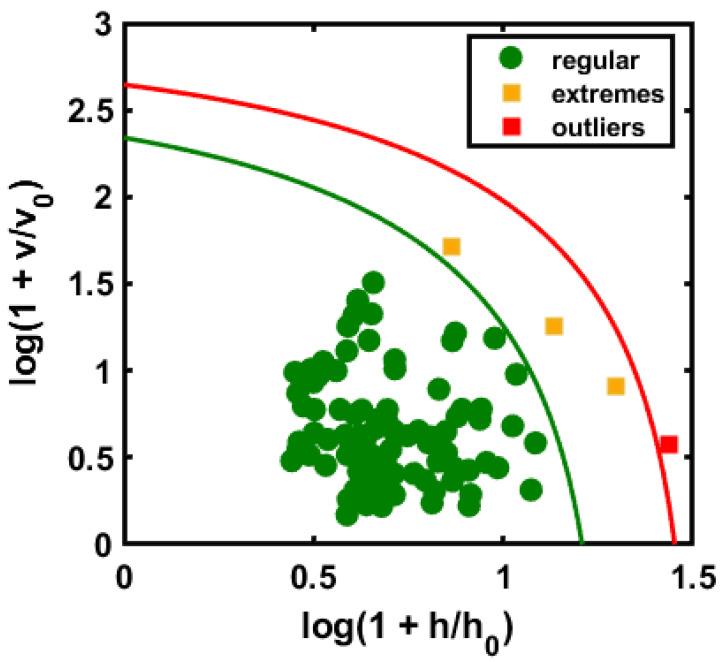
Chi-square acceptance area of DD-SIMCA for the target class.

Based on the above illustration of the DD-SIMCA application, the class model was first developed with 100 randomly selected pure almond samples. The initial task was to determine the appropriate number of factors to use in the model by examining the sensitivity (sensitivity of the model at which the lowest number of extreme and outliers noted) as a function of the number of factors added to it. Once the model was ready, the important task was to evaluate the model’s capability based on an unknown set of objects that were not used in the model construction. Table 2 summarizes the validation results for the model on the four sets of test data. The number of factors selected is based on type I errors in the model that results in the minimum number of model errors; the ultimate goal in selecting factors is the equality of data. Figure 5 presents the use of pure almond as a targeted class for model development based on chi-square analysis.

Each class-models present the acceptance area by determining the orthogonal vs. score distance, which can be represented by a given α-value. Figure 5 illustrates the chi-square acceptance area of DD-SIMCA for the target classes. The green curve in the plot defines the acceptance area (α = 0.01) for the pure samples, while the red curve limits the boundary for outlier acceptance (γ = 0.01). Each sample of the training set is described on such a plot by its position and is classified as either “regular”, a target group, or an “extreme”. The samples located outside the red line counted as outliers. In Figure 5, a single sample is located beyond the outlier boundary representing the γ-value. 

**Figure 6:**
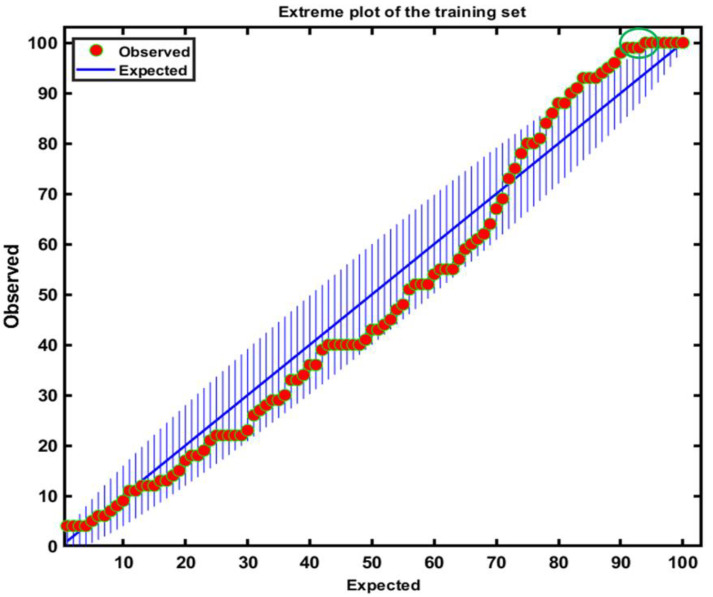
The extreme plot based on the developed model shows four samples deviating more significantly from the line (green circle).

Figure 6 represents an extreme plot useful to compare the extreme samples against the predicted ones. The extreme plot displays extreme and outliers which are segmented from the vertical lines. Furthermore, it can be used to assess the performance of the classification model for selecting the number of principal components. Figure 6 indicates that the consistency of four data points is slightly out of the tolerance area (circled in the green, upper right area of the figure).

**Figure 7:**
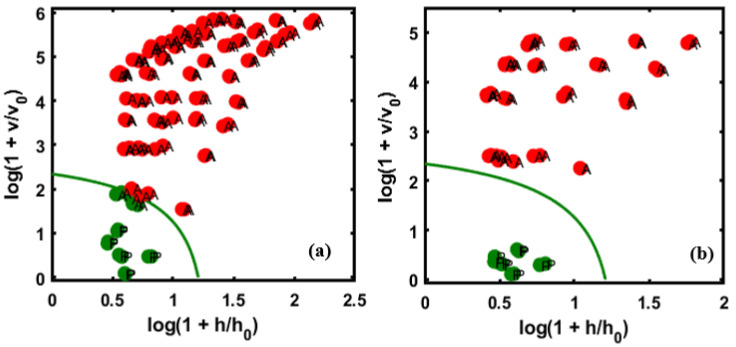
DD-SIMCA results for almond samples adulterated with apricot. Validation set-1 (**a**), and validation set-2 (**b**). The validation sets consisted of data from two different varieties of almond powder.

The sensitivity and specificity were calculated based on Equations (2) and (3), and the results are summarized in Table 2 for both training and test datasets. For the validation sets, Figure 7a,b show the almond powder adulterated with apricot for the first and second varieties, respectively, denoting the predictive performance of the DD-SIMCA chi-square acceptance area for the targeted class, where the P symbol represents a pure sample in the test set. Figure 7a indicates that five samples were misidentified as pure samples; however, there were no misclassifications in Figure 7b.

As observable from Figure 7a, five numbers of samples were misclassified in the non-targeted class corresponding to the 5% concentration of the data on the adulterated samples, and the acceptance area shows close relation with the remaining five samples related to the 5% group of samples. Meanwhile, there is no misclassification in the second validated set of data and the acceptance area shows a big gap between the target and non-targeted samples.

**Figure 8:**
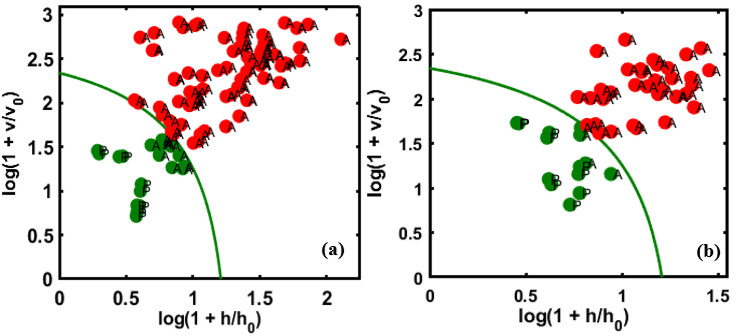
DD-SIMCA results for almond samples were adulterated with peanuts. Validation set-1 (**a**), and validation set-2 (**b**).

Additionally, the developed DD-SIMCA classification model was tested for discrimination of peanut-adulterated almond samples from the pure almond powder. Following the same strategy as for apricot adulterated almond samples, the model was validated with two different validation sets. The results indicated a higher number of misclassified samples in both validation sets. In particular, 11 adulterated samples were classified as pure in validation set-1 (Figure 8a), and 4 misclassified samples occurred for validation set-2 (Figure 8a).

The DD-SIMCA results for both almond-apricot adulteration and almond-peanut adulteration are summarized in Table 2. The performance of the DD-SIMCA model was determined based on sensitivity, specificity, and total classification accuracy (the percentage of total samples classified correctly). As shown in Table 2, the DD-SIMCA model classified apricot-adulterated almond samples with 96.5% accuracy for validation set-1 and 100% accuracy for validation set-2. However, comparatively lower accuracies were achieved for classification of peanut-adulterated almond samples, where 11 adulterated samples were misclassified as pure for validation set-1, and 4 samples for validation set-2. However, it should be noted that no pure almond powder sample was misclassified as adulterated in all cases, thus yielding 100% specificity.

According to the figures, in the first validation set (a), 11 samples were misclassified in the non-targeted groups, which correspond to 5% concentrations, Figure 8b indicates 4 samples that were misclassified compared to the first validation set. This misclassification might have resulted from the similarity in the spectral pattern of pure almond with 5% followed closely by the acceptance area and 7% concentration in the dataset.

Based on the data presented in Table 1, the highest accuracy was achieved for the validations datasets on the second variety of adulterated almond with apricot (100%) followed by the same adulterants in the first variety (95.5%). Meanwhile, the lowest accuracy (90% and 92%) was based on the varieties, respectively. However, few low adulterant concentration samples (5% and 7%) were misclassified as pure almond samples in both cases: Almond and apricot adulteration, and almond and peanut adulteration. Thus, it can be concluded that the DD-SIMCA model has the detection limit of over 7% adulteration.

### 3.3. Reproducibility with an External Validation Set

**Figure 9:**
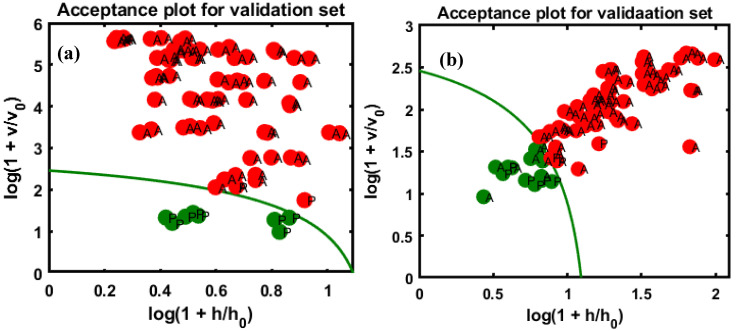
Predictive performance of the model based on the chi-square test determined by applying the DD-SIMCA method to the two unknown sets of data (external validation sets), (**a**) adulterated almond with apricot and (**b**) adulterated almond with peanuts.

With regards to the resulting models, contaminations with larger concentrations can be detected with a high sensitivity, thus the lower threshold of concentration detection is the limitation of the model. However, the result, also based on the feasibility study of point-based spectroscopy, demonstrated the same limitation regarding detection [31], i.e., concentrations below 7% were potentially not detected. The results were comparatively similar based on the FT-IR and FT-NIR analyses and slightly higher than the hyperspectral imaging results, with a 90–100% accuracy. The sensitivity of spectroscopic or spectral imaging systems can be mainly affected by two factors: Variation in illumination intensity and sensor responses. Specifically, hyperspectral imaging systems with a direct lighting system. Thus, these variations may reduce the performance of developed multivariate analysis models. Therefore, the calibration model should be tested with an external unknown validation data to justify the reproducibility of the calibration model [15,52]. For this reason, additional experimental data was imaged in two groups as a blind set that was not included in the calibrations set. The sample preparation for the external validation set was the same as for validation set-1 with slightly different adulterants’ concentration (0, 7, 15, 22, 30, 40, 45, and 50%) in both almond–apricot and almond–peanut adulterated samples. A total of 80 samples were extracted for each group (10 samples for each aforementioned concentration) and used as an external validation dataset to validate the DD-SIMCA calibration model. After performing the verification process, reasonably good results were obtained, with 97% total accuracy (specificity of 80%, and sensitivity of 100%) for almond–apricot adulteration as only two pure almond samples were misclassified as adulterated, as shown in Figure 9a. Meanwhile, adulterated almond–peanut shows comparatively lower accuracy as three pure samples were misclassified as adulterated and nine samples from 7% adulterant concentration were classified as pure almond samples (Figure 9b), thus attaining a total accuracy of 86% (specificity of 70%, and sensitivity of 87%).

According to the results, most of the misclassified adulterated samples related to the adulterated almond and peanuts belong to 7% concentrations. As previously discussed, the developed model has limitations for detecting low concentrations (<10%). However, the proposed method is confirmed to have good accuracy for detecting the almond powder authenticity even when the almond powder and adulterants are from different varieties.

Furthermore, a significant difference between the spectral data of almond varieties used in calibration and validations was confirmed by subjecting a total of 150 samples (50 samples from each variety of pure almond powder) for the Kruskal–Wallis test. These results suggest that despite being (statistically) significantly difference among the almond varieties, the DD-SIMCA technique has the potential to classify pure and adulterated almond powder with acceptable accuracy even when the calibration model is developed with different varieties of almond powder and is blindly validated with samples from different varieties measured at a different time with slightly different instrumental settings (i.e., illumination and sensor setting).

### 3.4. PLSR Model Development for Almond Adulterants

**Figure 10:**
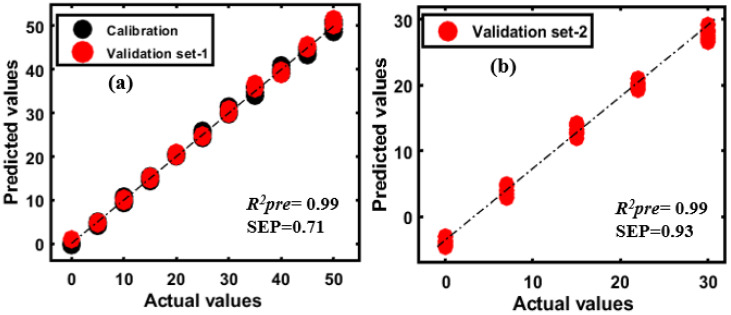
(**a**) PLSR prediction plots for apricot-adulterated almond (first variety); (**b**) results for the second variety of almond adulterated with apricot.

The extracted spectral data of all samples were arranged in a matrix (as previously discussed) and preprocessed with seven different pre-processing methods discussed in the spectral preprocessing section of this paper. The PLSR model was first developed for one variety of almond samples adulterated with either apricot or peanut and further validated with two different validation sets: Validation set-1 (consisting of adulterant with almond of the first variety), and validation set-2 (consisting of adulterant with almond of the second variety). The model performance is summarized in Table 3. Figure 10a shows the actual and predicted concentrations of apricot in almond powder (first variety) by the PLSR model developed with raw data (no preprocessing) for the calibration set and validation set-1. The PLSR yielded an *R*^2^*_pre_* of 0.99 with a SEP of 0.71%. Also, the model yielded a similar prediction accuracy and error when tested with validation set-2 (Figure 10b).

**Figure 11:**
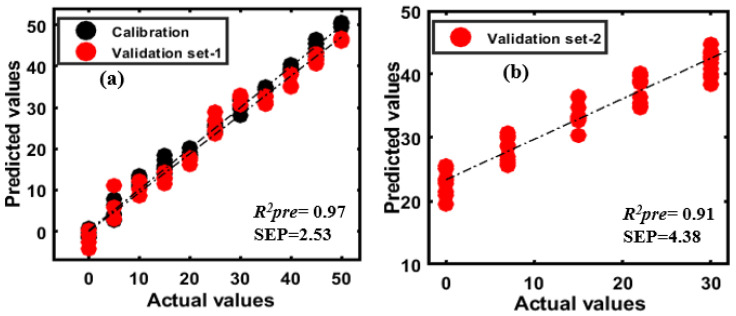
PLSR prediction plots (**a**) for peanut-adulterated almond (first variety); (**b**) results for the second variety of almond adulterated with peanut.

PLSR models using the spectral data of peanut-adulterated almond powder were developed in the same manner as for apricot adulterated almond powder. Among all preprocessing methods, SG-second yielded the best prediction accuracy with an *R*^2^*_pre_* of 0.97 and na SEP of 2.53% for validation set-1 (Figure 11a), while the model validated using the second set of data (validation set-2) resulted in poorer prediction, as shown in Figure 11b, with an *R*^2^*_pre_* of 0.91 and an SEP of 4.38%.

According to the resulting model, the performance of the PLSR model for almond adulterated with apricot corresponded better to all concentrations than almond adulterated with peanut. The main reason for the poorer performance of the second model invalidation based on the second variety of contaminated almonds is that the chemical composition bands for the contents of the almond and peanuts were virtually the same. Moreover, the obtained results from the PLSR model for validation set-2 are comparable with the results obtained using DD-SIMCA.

### 3.5. Spatial Distribution Maps of Adulterants in Almond Powder

**Figure 12:**
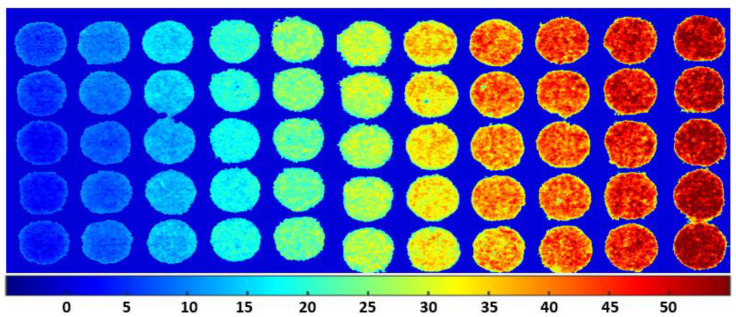
A chemical visualization map of almond is adulterated with apricot at different concentrations.

The HSI techniques for presenting the chemical contents of powdered samples represent a significant potential for better visualization of the composition of different samples, e.g., the detection and quantification of peanut traces in wheat flour through NIR hyperspectral imaging spectroscopy using principal component analysis [21] and the detection of peanut and walnut powders in whole wheat flour [18]. For this purpose, corrected hyperspectral imaging data were processed for the visualization of adulterant concentrations in almond powder in this study. Concentrations of almond powder are mapped with different apricot adulteration percentages in Figure 12. Before the development of the concentration mapped image, the background was eliminated from the samples using a simple threshold method, and the chemical images for each concentration were then generated using Equation (8). These images not only provided the spatial distribution of adulterations on the surfaces of samples but were also useful in determining the specific adulteration percentage mixed in with almond powder samples. The color scale from blue to red shown in the *x*-axis of the Figure 12 represents the concentration distribution ranging from 0–50%. Pure almond samples were used as a reference, while the adulterated percentage is based on color changes. From the resulting images, the adulterated samples have more intense color than those of the pure almond and become more intense (from blue to red) with increasing concentration of adulterant. The concentration images of the PLSR model mapped allow easy visualization of the adulterant concentration in almond powder based on the color intensity.

The image starts from dark blue (0%) to red color based on the adulterant percentage in the mixture. The last column of samples with 50% adulteration is dark red. In contrast, the chemical visualization map for the peanut-adulterated samples presented a weak distribution of chemical compositions on the surface of the samples owing to fewer differences within the spectral signature.

## 4. Conclusions

This study assessed the quality and quantity of adulterated almond powder using hyperspectral imaging combined with non-targeted classification analysis method. In order to develop a single calibration model to test an unknown collection of samples, the one-class DD-SIMCA classifier was used for the detection of potentially adulterated almond. Owing to the importance of equal quantitative analysis in terms of quality analysis of products, an attempt was made to extend the model to different varieties of almond powder using PLSR. The tested model potentially predicted the added adulterants in the different varieties of apricot with higher accuracy but relatively low performance in the case of peanut adulteration. A chemical visualization map related to the composition of each concentration was then developed. The obtained results demonstrated the potential of hyperspectral imaging coupled with different chemometric methods for quality and quantity authentication of adulterated powder samples. Although the potential of the developed chemometric models was tested for two different varieties of almond powder samples, the classification models can continue to be updated by adding more spectral data from different varieties, origin, and different timely stored almond powder.

## Figures and Tables

**Table 1:** Summary of the descriptive statistics for the calibration and validation datasets used in the data-driven soft independent modeling of class analogy (DD-SIMCA) and partial least square regression (PLSR) analyses (excluding the number of samples, all units are in %).

		Number of Samples	Maximum	Minimum	Mean ± SD
DD-SIMCA	Calibration	100	0	0	0 ± 0
	Validation1	110	50	0	25.04 ± 15.94
	Validation2	50	30	0	14.8 ± 10.72
PLSR	Calibration	66	50	0	25 ± 15.94
	Validation1	44	50	0	25 ± 15.99
	Validation2	44	30	0	13.45 ± 11.07

**Table 2:** DD-SIMCA classification results were obtained for validation datasets. (AA: Almond and apricot; AP: Almond and peanut).

	Sensitivity (%)	Number of Correctly Classified Samples/Total Number of Samples	Specificity (%)	Number of Correctly Classified Samples/Total Number of Pure Samples	Accuracy (%)
Val-1st-AA	95	95/100	100	10/10	95.5
Val-2nd-AA	100	40/40	100	10/10	100.0
Val-1st-AP	89	89/100	100	10/10	90.0
Val-2nd-AP	90	36/40	100	10/10	92.0

**Table 3:** Presents the results obtained from the developed model with the first variety of almonds when used to predict the second variety of adulterated almond for the four groups of data, which were pre-processed with various techniques. The SG-2nd derivatives provided the best predictive result for both varieties.

Pre-Processing Methods	1st Variety Adulterated with Apricot				2nd Variety Adulterated with Apricot		1st Variety Adulterated with Peanut				2nd Variety Adulterated with Peanut	
	*R* ^2^ *cal*	*SEC* (%)	*R* ^2^ *_pre_*	*SEP* (%)	*R* ^2^ *_pre_*	*SEP* (%)	*R* ^2^ *cal*	*SEC* (%)	*R* ^2^ *_pre_*	*SEP* (%)	*R* ^2^ *_pre_*	*SEP* (%)
Mean Normalization	0.99	1.16	0.98	1.92	0.99	1.42	0.99	1.83	0.83	6.56	0.76	5.21
Max Normalization	0.99	0.76	0.99	1.00	0.99	0.80	0.98	2.20	0.81	7.05	0.75	5.35
Range Normalization	0.99	1.53	0.98	2.13	0.96	10.9	0.95	3.45	0.82	6.65	0.76	5.32
MSC	0.99	1.28	0.98	1.81	0.98	2.28	0.98	2.17	0.75	8.21	0.56	7.07
SNV	0.99	0.85	0.99	1.16	0.98	4.11	0.98	2.17	0.75	8.16	0.56	7.13
SG 1st Derivatives	0.99	0.79	0.99	0.95	0.98	1.38	0.99	1.68	0.98	2.18	0.94	4.77
SG 2nd Derivatives	0.99	0.71	0.99	0.71	0.99	0.93	0.99	1.48	0.97	2.53	0.91	4.38
Raw	0.99	0.75	0.99	0.87	0.99	1.10	0.98	1.00	0.98	2.40	0.90	5.71
